# Discordant financial conflicts of interest disclosures between clinical trial conference abstract and subsequent publication

**DOI:** 10.7717/peerj.6423

**Published:** 2019-02-11

**Authors:** Glen J. Weiss, Roger B. Davis

**Affiliations:** 1 Department of Medicine, Beth Israel Deaconess Medical Center, Boston, MA, USA; 2 Department of Medicine, Harvard Medical School, Boston, MA, USA

**Keywords:** Financial conflicts of interest, Clinical trials, Oncology, Discordance, Publication

## Abstract

**Background:**

Financial conflicts of interest (FCOI) are known to be prevalent in medicine. Authorship of pivotal trials reap non-financial benefits including publication productivity that can be used for assessment of tenure positions and promotion. The purpose of this investigation was to quantify the prevalence and discordance of academic trial author (authors) FCOI in industry-sponsored drug trials that were initially presented as oral abstracts and subsequently resulted in a peer-reviewed publication.

**Methods:**

Oral abstracts from the American Society of Clinical Oncology (ASCO) 2017 Annual Meeting that were subsequently published were identified. Studies that were non-industry sponsored, non-adult, or non-therapeutic trials were excluded. Studies that did not have a subsequent peer-reviewed publication or had a publication preceding the ASCO 2017 Annual Meeting were also excluded. FCOI was categorized and impact factor (IF) for the journal at the time of publication was retrieved. FCOI discordance between the oral abstract and publication was calculated based on geographic location and IF.

**Results:**

A total of 22 paired abstract and publications met inclusion criteria for further analysis. A total of 384 authors were identified, of these 280 authors (74.1%) were included in both the oral abstract and subsequent publication. A total of 76% of these 280 authors had FCOI and 66.4% had FCOI discordance. There were statistically significant differences for the sum of FCOI discordance for U.S.-based authors (*p* = 0.0004) but not for journal IF. When analyzing the sum of absolute differences of FCOI discordance, statistical significance was reached for authors from any of the three geographic regions, as well as, low and high IF journals (all *p*-values < 0.0001).

**Conclusions:**

This study draws attention to the lack of uniformity and vetting of FCOI reporting in abstracts and journals publishing solid tumor oncology trial results. This is particularly concerning, since FCOI is prevalent globally.

## Introduction

Financial conflicts of interest (FCOI) are known to be prevalent in oncology, including potential influence on conclusions of seminal clinical trials (Hampson et al., 2007; Johnston & Go, 2007), cost-effectiveness studies (Jang, Chae & Majhail, 2011), practice guideline panels (Tibau et al., 2015; Mitchell, Basch & Dusetzina, 2016; Daly, Bach & Page, 2018), Food and Drug Administration (FDA) advisory committees (Lurie et al., 2006; Hayes & Prasad, 2018), social media (Tao et al., 2017), and journal editors (Kaestner & Prasad, 2017). A recent article reported that 32% of 344 U.S. based oncologist authors did not fully disclose their payments from the trial sponsor in the publication of a pivotal clinical trial that led to U.S. FDA approval (Wayant et al., 2018). In that same study, 76.5% of oncologist authors received at least one industry payment. Authorship of pivotal trials reap non-financial benefits including publication productivity that can be used for assessment of tenure positions and promotion (Tarkang, Kweku & Zotor, 2017). FCOI prevalence is not limited to just academic trial authors (authors) and publications, but can be found amongst participants of FDA Advisory Committee meetings (Hayes & Prasad, 2018) and hematologist oncologists that post on social media platforms (Tao et al., 2017). Discordance in reporting of FCOI has been blamed in part by non-uniform reporting requirements across journals (Shawwa et al., 2016).

The primary objective of this investigation was to quantify the prevalence of author FCOI in industry-sponsored drug trials that were initially presented as oral abstracts and subsequently resulted in a peer-reviewed publication. The secondary objective was to identify the number of FCOI discordance amongst the authors, including examining geographic region of the authors and the journal’s impact factor (IF).

## Materials and Methods

**Figure 1:**
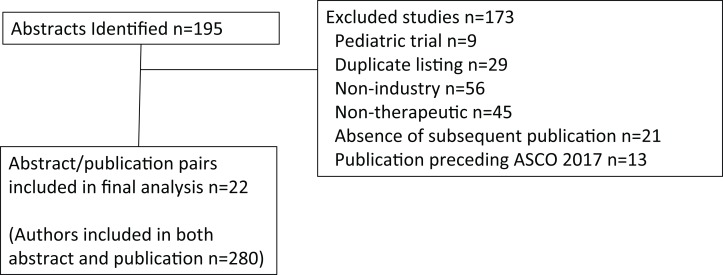
CONSORT diagram.

Oral abstracts covering solid tumors presented at the American Society of Clinical Oncology (ASCO) 2017 Annual Meeting were identified (*n* = 195) by electronic search on abstracts.asco.org, using search term Type: Oral abstract session and Topic: Cancers (each sub-topic was selected except for Hematologic malignancies). From this search list, each abstract identified was then manually reviewed for relevance and inclusion. The review was conducted from August 13 to August 22, 2018, and excluded non-industry sponsored studies (*n* = 56), non-therapeutic trials (*n* = 45), duplicate listings (*n* = 29), the absence of a subsequent peer-reviewed publication (*n* = 21), a corresponding study publication preceding the ASCO 2017 Annual Meeting (*n* = 13), or pediatric cancer studies (*n* = 9) (Fig. 1). Note that abstracts could have been identified by more than one category, but were included only if the trial was an industry sponsored therapeutic trial in adult cancers and subsequently resulted in a peer-reviewed publication and the results had not been published in peer reviewed literature prior to the ASCO 2017 Annual Meeting. FCOI were grouped according to the ASCO FCOI categories. However, in order to limit small numbers of rare FCOI, leadership role and patent were grouped together, and expert testimony was grouped with consulting or advisory role. Thus, for the abstract and separately for the publication, each unique FCOI falling into these categories were tallied for leadership role or patent, stock or ownership interest, honoraria, consulting or advisory board or expert testimony, speaker’s bureau, research funding, travel support, or other for each author. An author was included in analysis and defined as those having a primary affiliation with an oncology clinic, cancer center, or university listed in the publication. Authors listed more than once were treated separately for each oral abstract/publication pairing that met all other inclusion criteria (File S1). Authors were either based in the U.S., Europe, or rest of world (ROW) according to the geographic location of their institutional affiliation. The 2017 IF for the journals was retrieved from the Journal Citation Reports 2018 release (https://clarivate.com/blog/tag/jcr-2018/).

The difference between the number of conflicts reported in the ASCO abstract and the final publication for each category of conflict was calculated. The sum for each author across all categories was calculated. The sum of the absolute value of the differences was also calculated. The average using a generalized estimating equation model to account for likely correlation among authors on the same abstract was estimated. Additional models were fit to estimate the average difference in each region and for low-impact (below the median IF) and high-impact journals (above the median IF). Estimated means and 95% confidence intervals, and *p*-values testing whether the means are equal to zero were reported. To adjust for multiple testing, a Bonferroni procedure was used and a *p*-value less than 0.0042 was considered to be statistically significant. The estimates of the mean and 95% confidence intervals for each category of conflict using the same modeling approach were also reported, but statistical tests for these outcomes were not conducted. Analyses were performed using SAS Statistical Software (version 9.4 for Windows; SAS Institute, Cary, NC, USA). This study was an investigation of publicly available reports which is exempt from institutional review board approval.

## Results

Overall, 22 unique clinical trial oral abstract/publication pairs met the inclusion criteria. A total of 378 authors were identified, of these 280 authors (74.1%) were included in both the oral abstract and subsequent publication. Of these 280 authors, no FCOI was declared by 12 U.S.-based, 21 Europe-based, and 33 ROW-based authors. When excluding research funding, which may be direct to the institution, no other FCOI was declared by a total of 86 of 280 authors (30.7%). A total of 18 authors had more than oral abstract/publication pair meeting the inclusion criteria. Table 1 shows oral abstract and publication FCOI by geographic region and category. Table 2 lists the journal and its IF publishing the included trials, along with the discordance between the oral abstract/publication pairs. Of the 280 authors, 186 (66.4%) had FCOI discordance in reporting. The mean FCOI discordance was 7.3 (minimum–maximum 0–26) for U.S. authors (*n* = 104), 5.6 (minimum–maximum 0–24) for European authors (*n* = 97), and 4.1 (minimum–maximum 0–21) for ROW (*n* = 79). Evaluating FCOI discordance of two standard deviations above the mean for the entire author population (a FCOI discordance > 20.9), there were 14 authors, seven from the U.S., four from Europe, and three from the ROW (minimum–maximum 21–38). There were 55 authors with a FCOI discordance greater than 10. Four of those 55 authors had more than one oral/abstract/publication included in this analysis, but only one of their FCOI discordances was greater than 10. There were statistically significant differences for the sum of FCOI discordance for U.S.-based authors (*p* = 0.0004) but not for journal IF categories. When analyzing the sum of absolute differences of FCOI discordance, statistical significance was reached for authors from any of the three geographic regions, as well as low and high IF journals (all *p*-values < 0.0001). The estimates of the mean and 95% confidence intervals for each category of conflict can be found in the Supplementary Materials.

**Table 1:** Oral abstract and publication FCOI by geographic region and category.

	Geographic region	# Authors	# of leadership or patent	# of stocks or ownership interest	# of honoraria	# of consulting or advisory	# speaker’s bureau	# of research funding	# of travel	Other
Abstract	US	104	18	29	101	453	49	458	54	21
	Europe	97	1	4	145	269	85	128	94	1
	ROW	79	3	2	134	162	14	125	27	0
Publication	US	104	5	11	88	345	31	248	41	2
	Europe	97	1	4	137	297	50	159	63	2
	ROW	79	1	1	74	139	0	119	12	0

**Notes:**

# = number.

ROW, rest of world.

**Table 2:** Journal carry the clinical trial publication and its IF and FCOI discordance.

Journal	IF	Δ Leadership or patent	Δ Stock or ownership interest	Δ Honoraria	Δ Consulting or advisory	Δ Speaker’s bureau	Δ Research funding	Δ Travel	Δ Other
Cancer chemotherapy and pharmacology	2.808	0	0	22	19	6	36	8	0
Clinical cancer research	10.199	0	0	0	2	3	4	2	0
JAMA oncology	20.871	0	0	29	24	4	17	3	0
Lancet respiratory medicine	21.466	0	0	28	37	4	17	6	0
Cancer discovery	24.373	3	12	3	90	0	82	2	11
Journal of clinical oncology (six of the publications)	26.303	9	9	37	64	14	37	35	0
Nature medicine	32.621	0	0	30	33	6	26	4	10
Lancet oncology (five of the publications)	36.421	2	4	90	150	47	107	53	1
JAMA	47.661	0	0	0	4	0	0	0	0
Lancet	53.254	0	0	23	37	11	28	6	0
New England Journal of medicine (three of the publications)	79.260	5	6	46	83	12	111	12	0

**Notes:**

IF, impact factor; FCOI, financial conflicts of interest.

Delta symbol = FCOI discordance between abstract and publication.

## Discussion

Financial conflicts of interest can influence how the results of a trial are presented (Hampson et al., 2007; Tibau et al., 2015; Kaestner & Prasad, 2017) at conferences and/or peer-reviewed publications, which are often broadcast in various media to other professionals and the general public. FCOI has been shown to influence economic analyses about industry sponsored products (Jang, Chae & Majhail, 2011). Authors with FCOI with the industry sponsor marketing a drug have been observed to more frequently endorse that specific pharmaceutical (Tibau et al., 2015). While Centers for Medicare and Medicaid Services (CMS) Open Payments (https://www.cms.gov/openpayments) allows for deeper interrogation of FCOI for U.S.-based authors, there is no publicly searchable tool for FCOI of non-U.S.-based authors. Perhaps not surprising, like U.S.-based authors, authors from other countries have a similar mean number of FCOIs. Like Wayant et al. (2018) this study observed FCOI discordances between two sources for the same clinical trial. Time interval alone between abstract and subsequent publication is unlikely to be the only factor, since the maximum interval was less than 15 months. Examination of the sum of the absolute differences for FCOI discordance revealed that regardless of author location or the journal IF, FCOI reporting between an oral abstract and its subsequent peer-reviewed publication is significantly different. More often, the oral abstract FCOI discordance in lower IF journals might be due to less stringent FCOI reporting requirements. Recently, failure to appropriately report FCOI led to the resignation of a prominent oncologist (Thomas & Ornstein, 2018). In this study, that same oncologist had a FCOI discordance of 10. Further, 55 additional authors were identified with FCOI discordance greater than 10.

This study has several limitations. First, FCOI declaration, while mandatory, is self-reported and not verified. These declarations do not identify how they may affect the authors interpretation or dissemination of the research results. Second, because the majority of authors in this analysis are not based in the U.S., monetary values cannot be readily assessed with FCOI declarations. Third, ideally the extraction and analyses of discordance would be performed by more than one investigator.

Potential remedies for minimizing FCOI discordance would be to either adopt uniform reporting policies across conferences and journals (e.g., ICMJE COI form) or doing away with self-disclosure and providing a link to the CMS Open Payments database for the author. A limitation of the link would be that it does not cover non-U.S.-based authors.

## Conclusions

In concordance with the press investigation of failure to properly disclose FCOI, this study draws attention to the lack of uniformity and vetting of FCOI reporting in abstracts and journals publishing solid tumor oncology trial results. This is particularly concerning, since FCOI is prevalent globally, ranging from 58.2% to 88.5% amongst authors. One solution to minimize FCOI discordance would be to either adopt uniform reporting policies across conferences and journals.

## Supplemental Information

10.7717/peerj.6423/supp-1 Supplemental Information 1 Additional rules applied to categorization of FCOI.

10.7717/peerj.6423/supp-2 Supplemental Information 2 Raw data of all authors identified. Rows with red highlighted cells—industry author, no location or FCOI data not entered.
N/A- disclosure not available for abstract because author was added after abstract presentation.
N/A- disclosure not available for abstract because author was removed in the subsequent publication.

10.7717/peerj.6423/supp-3 Supplemental Information 3 Estimate of the Means. Bold text are the conditions, green highlights are categories for sum of all discordances and sum of the absolute value of all discordances that were statistically significant.

## Additional Information and Declarations

### Data Availability

The following information was supplied regarding data availability: Raw data is available in the Supplemental Files.
