# Supplementary material for: Discordant financial conflicts of interest disclosures between clinical trial conference abstract and subsequent publication
Source: PeerJ. 2019 Feb 11;7:e6423. doi: 10.7717/peerj.6423 (PMC6375255; doi:10.7717/peerj.6423)
Supplement: Supplemental Information 1 [file peerj-07-6423-s001.docx]

Rules for categorization from peer-reviewed publications

1. Same categories as ASCO abstract, counted and put into same category
2. If employment listed for an academic trial author, this was not counted so long as employment was not with industry/pharmaceutical company
3. Industry authors criteria was met for exclusion of this analysis if employment or affiliation with industry/pharmaceutical company was disclosed.
4. If an author listed the same company name more than once for the same COI category, that company was counted just once
5. Patents or pending patents or royalties were added to leadership COI due to small numbers, while expert testimony was added to consulting/advisory COI due to small numbers.
6. Travel or expenses that were not associated with any company were not counted. This was also checked for consistency in that author’s declaration in the abstract and there was no discordance.
7. Any COI that is the author’s institution or employer that was declared by the author was not counted. Example: Omsk Clinical Oncology Dispensary provided travel was counted as zero for travel.
8. Grants or non-financial support from industry were counted as research funding COI
9. Personal fees were typically itemized in the peer-reviewed publication and counted as consulting/advisory board COI unless explicitly stated otherwise.
10. Advisory board and speaking COI were counted as separately as consulting/advisory and speaker’s bureau COI, respectively.
11. Advisory board honorarium payable to institution was counted separately as consulting/advisory and honorarium COI, respectively.
12. Stock to spouse or author owned stock was counted as stock/ownership COI.
13. Investigator initiated trials were counted as research funding COI.
14. Scientific advisory boards were counted as consulting/advisory COI.
