# Supplementary material for: Discordant financial conflicts of interest disclosures between clinical trial conference abstract and subsequent publication
Source: PeerJ. 2019 Feb 11;7:e6423. doi: 10.7717/peerj.6423 (PMC6375255; doi:10.7717/peerj.6423)
Supplement: Supplemental Information 3 — Bold text are the conditions, green highlights are categories for sum of all discordances and sum of the absolute value of all discordances that were statistically significant. [file peerj-07-6423-s003.docx]

**Supplementary File. Estimate of the Means**

**Sum of all discordances**

Analysis Of GEE Parameter Estimates

Empirical Standard Error Estimates

Standard 95% Confidence

Parameter Estimate Error Limits Z Pr > |Z|

Intercept 0.0000 0.0000 0.0000 0.0000 . .

author_location__US_ ROW 1.6926 0.8000 0.1246 3.2605 2.12 0.0344

author_location__US_ europe 0.1498 0.9765 -1.7641 2.0637 0.15 0.8781

author_location__US_ usa 3.5801 1.0022 1.6158 5.5444 3.57 0.0004

Analysis Of GEE Parameter Estimates

Empirical Standard Error Estimates

Standard 95% Confidence

Parameter Estimate Error Limits Z Pr > |Z|

Intercept 0.0000 0.0000 0.0000 0.0000 . .

highimpact no 4.7639 1.6760 1.4791 8.0488 2.84 0.0045

highimpact yes 0.9768 0.5404 -0.0824 2.0360 1.81 0.0707

**Sum of absolute values of all discordances**

Analysis Of GEE Parameter Estimates

Empirical Standard Error Estimates

Standard 95% Confidence

Parameter Estimate Error Limits Z Pr > |Z|

Intercept 0.0000 0.0000 0.0000 0.0000 . .

author_location__US_ ROW 4.7494 1.1905 2.4160 7.0828 3.99 <.0001

author_location__US_ europe 5.7894 0.9627 3.9025 7.6762 6.01 <.0001

author_location__US_ usa 6.6187 0.9792 4.6995 8.5379 6.76 <.0001

Analysis Of GEE Parameter Estimates

Empirical Standard Error Estimates

Standard 95% Confidence

Parameter Estimate Error Limits Z Pr > |Z|

Intercept 0.0000 0.0000 0.0000 0.0000 . .

highimpact no 6.5421 1.6243 3.3586 9.7256 4.03 <.0001

highimpact yes 5.5938 0.9002 3.8295 7.3581 6.21 <.0001

**Leadership COI or patent**

Analysis Of GEE Parameter Estimates

Empirical Standard Error Estimates

Standard 95% Confidence

Parameter Estimate Error Limits Z Pr > |Z|

Intercept 0.0000 0.0000 0.0000 0.0000 . .

author_location__US_ ROW 0.0352 0.0207 -0.0053 0.0758 1.70 0.0886

author_location__US_ europe 0.0023 0.0073 -0.0120 0.0166 0.32 0.7490

author_location__US_ usa 0.1276 0.0751 -0.0197 0.2749 1.70 0.0894

Analysis Of GEE Parameter Estimates

Empirical Standard Error Estimates

Standard 95% Confidence

Parameter Estimate Error Limits Z Pr > |Z|

Intercept 0.0000 0.0000 0.0000 0.0000 . .

highimpact no -0.0140 0.0122 -0.0379 0.0099 -1.15 0.2509

highimpact yes 0.0817 0.0423 -0.0011 0.1646 1.93 0.0532

**Stock/Ownership**

Analysis Of GEE Parameter Estimates

Empirical Standard Error Estimates

Standard 95% Confidence

Parameter Estimate Error Limits Z Pr > |Z|

Intercept 0.0000 0.0000 0.0000 0.0000 . .

author_location__US_ ROW 0.0229 0.0168 -0.0099 0.0558 1.37 0.1715

author_location__US_ europe 0.0004 0.0191 -0.0369 0.0378 0.02 0.9823

author_location__US_ usa 0.1488 0.1014 -0.0499 0.3475 1.47 0.1422

Analysis Of GEE Parameter Estimates

Empirical Standard Error Estimates

Standard 95% Confidence

Parameter Estimate Error Limits Z Pr > |Z|

Intercept 0.0000 0.0000 0.0000 0.0000 . .

highimpact no 0.1672 0.1457 -0.1184 0.4528 1.15 0.2513

highimpact yes 0.0286 0.0300 -0.0301 0.0873 0.96 0.3396

**Honoraria**

Analysis Of GEE Parameter Estimates

Empirical Standard Error Estimates

Standard 95% Confidence

Parameter Estimate Error Limits Z Pr > |Z|

Intercept 0.0000 0.0000 0.0000 0.0000 . .

author_location__US_ ROW 0.7930 0.3735 0.0611 1.5250 2.12 0.0337

author_location__US_ europe -0.0792 0.4428 -0.9470 0.7886 -0.18 0.8580

author_location__US_ usa 0.1536 0.1493 -0.1390 0.4462 1.03 0.3036

Analysis Of GEE Parameter Estimates

Empirical Standard Error Estimates

Standard 95% Confidence

Parameter Estimate Error Limits Z Pr > |Z|

Intercept 0.0000 0.0000 0.0000 0.0000 . .

highimpact no 0.4279 0.3801 -0.3171 1.1728 1.13 0.2603

highimpact yes 0.1987 0.2221 -0.2366 0.6341 0.89 0.3709

**Consulting/Advisory or expert testimony**

Analysis Of GEE Parameter Estimates

Empirical Standard Error Estimates

Standard 95% Confidence

Parameter Estimate Error Limits Z Pr > |Z|

Intercept 0.0000 0.0000 0.0000 0.0000 . .

author_location__US_ ROW 0.2996 0.5175 -0.7146 1.3139 0.58 0.5626

author_location__US_ europe -0.2200 0.2991 -0.8063 0.3663 -0.74 0.4620

author_location__US_ usa 0.9194 0.5334 -0.1261 1.9649 1.72 0.0848

Analysis Of GEE Parameter Estimates

Empirical Standard Error Estimates

Standard 95% Confidence

Parameter Estimate Error Limits Z Pr > |Z|

Intercept 0.0000 0.0000 0.0000 0.0000 . .

highimpact no 1.8188 0.8042 0.2426 3.3949 2.26 0.0237

highimpact yes -0.1091 0.2123 -0.5252 0.3070 -0.51 0.6073

**Speaker’s bureau**

Analysis Of GEE Parameter Estimates

Empirical Standard Error Estimates

Standard 95% Confidence

Parameter Estimate Error Limits Z Pr > |Z|

Intercept 0.0000 0.0000 0.0000 0.0000 . .

author_location__US_ ROW 0.1787 0.0748 0.0321 0.3253 2.39 0.0169

author_location__US_ europe 0.3510 0.1911 -0.0236 0.7257 1.84 0.0663

author_location__US_ usa 0.1711 0.0907 -0.0066 0.3488 1.89 0.0592

Analysis Of GEE Parameter Estimates

Empirical Standard Error Estimates

Standard 95% Confidence

Parameter Estimate Error Limits Z Pr > |Z|

Intercept 0.0000 0.0000 0.0000 0.0000 . .

highimpact no 0.1615 0.0623 0.0394 0.2836 2.59 0.0095

highimpact yes 0.2593 0.0998 0.0637 0.4549 2.60 0.0094

**Research Funding**

Analysis Of GEE Parameter Estimates

Empirical Standard Error Estimates

Standard 95% Confidence

Parameter Estimate Error Limits Z Pr > |Z|

Intercept 0.0000 0.0000 0.0000 0.0000 . .

author_location__US_ ROW 0.0726 0.2573 -0.4317 0.5769 0.28 0.7779

author_location__US_ europe -0.2436 0.3567 -0.9428 0.4556 -0.68 0.4947

author_location__US_ usa 1.9323 0.5925 0.7711 3.0935 3.26 0.0011

Analysis Of GEE Parameter Estimates

Empirical Standard Error Estimates

Standard 95% Confidence

Parameter Estimate Error Limits Z Pr > |Z|

Intercept 0.0000 0.0000 0.0000 0.0000 . .

highimpact no 1.9710 0.8136 0.3764 3.5656 2.42 0.0154

highimpact yes 0.2408 0.2205 -0.1913 0.6729 1.09 0.2747

**Travel**

Analysis Of GEE Parameter Estimates

Empirical Standard Error Estimates

Standard 95% Confidence

Parameter Estimate Error Limits Z Pr > |Z|

Intercept 0.0000 0.0000 0.0000 0.0000 . .

author_location__US_ ROW 0.2461 0.1205 0.0098 0.4824 2.04 0.0412

author_location__US_ europe 0.2656 0.2453 -0.2152 0.7464 1.08 0.2789

author_location__US_ usa 0.1489 0.0969 -0.0410 0.3388 1.54 0.1244

Analysis Of GEE Parameter Estimates

Empirical Standard Error Estimates

Standard 95% Confidence

Parameter Estimate Error Limits Z Pr > |Z|

Intercept 0.0000 0.0000 0.0000 0.0000 . .

highimpact no 0.1279 0.1057 -0.0793 0.3352 1.21 0.2263

highimpact yes 0.2440 0.1312 -0.0131 0.5012 1.86 0.0629
